# The prevalence of hookah smokers among Belarussian children and adolescents

**DOI:** 10.18332/tpc/159488

**Published:** 2023-02-21

**Authors:** Tanya Pronina, Sergei Sychik

**Affiliations:** 1 Republican Scientific and Practical Centre of Hygiene, Minsk, Belarus

**Keywords:** hookah smoking, waterpipe, prevalence, children and adolescents

## Abstract

**INTRODUCTION:**

The purpose of this survey was to assess the prevalence of hookah smokers among children and adolescents aged 12–16 years in Belarus.

**METHODS:**

This survey involved 3485 people, including 1737 girls and 1726 boys, and included 6 questions from the main questionnaire of the GYTS aimed at hookah consumption. The statistical processing was performed using the SUDAAN software package; a 95% confidence interval was calculated to calculate weighted prevalence estimates and standard errors.

**RESULTS:**

According to the GYTS carried out in the Republic of Belarus in 2021, hookah smoking is becoming increasingly popular. The number of adolescents who have ever smoked a hookah is 9.3%. The prevalence of regular hookah smokers is quite low (0.9%). But there are justified fears that teens who have never smoked cigarettes but have tried hookah are more likely to start smoking cigarettes and become regular cigarette smokers two years later.

The frequency of hookah use naturally increases with age. The number of adolescents who have ever smoked a hookah among those aged 14, 15 and 16 years (10.7%, 11.7% and 18.2%, respectively), was higher than among students aged 12 years (5.5%) or 13 years (5.6%) (p<0.001); hookah smoking was higher among students in the 9th and 10th grades (13.0% and 14.2%, respectively), then among students in the 7th grade (5.1%) (p<0.001).

**CONCLUSIONS:**

This national survey provides the first data of prevalence of hookah smoking among children and adolescents in Belarus and allows us to conclude that the growing popularity of waterpipe tobacco smoking dictates the need for timely recommendations on waterpipe smoking policy.

## INTRODUCTION

The American Lung Association’s 2022 State of Tobacco Control annual report assesses the effectiveness of tobacco control laws and policies and recognizes the progress made in reducing tobacco use over the past 20 years. However, flavored tobacco products for young people remain a major threat to future progress^1^. Hookah smoking is gaining popularity nationwide, especially among urban youth, and Belarussian children are no exception. At least 82 toxic chemicals and carcinogens have been identified in hookah smoke^2,3^. In addition, many hookah smokers consider the practice less harmful than smoking cigarettes^4^. One of the reasons for this growing popularity is the fact that waterpipe tobacco contains child-friendly flavors such as watermelon, tropical fruit, orange cream, caramel, chocolate, tutti frutti, vanilla, strawberry and others, which help mask the harshness of smoking.

Hookah smoking is a growing threat to public health as it is recognized to be a highly social activity during which hookah users smoke tobacco filtered through a waterpipe that is often shared by the group. A systematic review of studies on the prevalence of waterpipe smoking in various populations showed an alarmingly high number of adherents among high school students. In the Eastern Mediterranean region, the highest prevalence of waterpipe use in the world among children aged 13–15 years varies from 9% to 15% of the range of waterpipe smoking^5^. In other World Health Organization regions, waterpipe tobacco smoking is less common than cigarette smoking.

One hookah filling contains 6.25 mg of nicotine, while 1 cigarette contains up to 1.5 mg of nicotine. While a regular cigarette requires from 8 to 10 puffs, an hour-long hookah smoking session can include up to 200 puffs, potentially exposing the smoker to more smoke for a longer period^4^. Short-term hookah use is associated with acute health effects, including increased heart rate and blood pressure, decreased lung function, and carbon monoxide poisoning. Long-term effects include impaired lung function, chronic obstructive pulmonary disease, esophageal and gastric cancer, and the risk of low birth weight and congenital malformations.

There is an absence of nationally representative prevalence studies of waterpipe tobacco use and dual use with other tobacco products in young people in Belarus. Hence, this survey aimed to assess the prevalence of hookah smokers among children and adolescents aged 12–16 years in Belarus.

## METHODS

An analysis of the GYTS, a nationally representative cross-sectional study of 3485 students aged 13–15 years of 39 participating schools in Belarus was conducted in 2021, including optional hookah/nargille/waterpipe smoking questions. The study used a two-stage selection of schoolchildren in grades 7–9 to conduct the survey. At the 1st stage, information was collected about all schools of the republic, where children of the target group in the study schools in which the survey was subsequently conducted, were selected by the statistical sampling method according to the number of students in grades 7– 10. In the 2nd stage, the grades whose students participated in the questionnaire were selected by random sampling in each school. A pre-tested modified GYTS questionnaire was used at school. This survey involved 3485 students in Belarus, including 1737 girls and 1726 boys. The school response rate was 100.0%, the student response rate was 81.2%, and the overall response rate was 81.2%.

The survey includes 6 questions from the main Questionnaire of the GYTS aimed at hookah consumption: 1) ‘Have you ever tried or experimented with any form of smoked tobacco products other than cigarettes?’; 2) ‘During the past 30 days, did you use any form of smoked tobacco products other than cigarettes (such as pipes, cigars, mini-cigars, hookah/nargile/waterpipe)?’; 3) ‘Have you ever tried or experimented with smoking hookah or nargille?’; 4) ‘During last 30 days, on how many days did you smoke hookah or nargille?’; 5) ‘Do you want to stop smoking hookah or nargille now?’; and 6) ‘Do you think smoking hookah or nargille is harmful to your health?’.

The completed answer sheets were sent to the US CDC where data were entered and analyzed using Epi Info. The statistical processing was performed using the SUDAAN software package. A p<0.05 was considered significant, and the results were expressed as odds ratio (OR) with a 95% confidence interval (CI).

## RESULTS

According to the GYTS carried out in the Republic of Belarus in 2021, the number of adolescents who have ever smoked a hookah is 9.3% (Table 1). The prevalence of regular hookah smokers is quite low (0.9%). The frequency of hookah use increased with age. The number of adolescents who have ever smoked a hookah among students aged 14, 15 and 16 years (10.7%, 11.7% and 18.2%, respectively), was higher than among students aged 12 years (5.5%) or 13 years (5.6%) (p<0.001).

**Table 1:** Prevalence of hookah use and attitudes towards it, among students by age, class and gender, GYTS Belarus, 2021

*Variable*	*Gender*			*Grade*				*Age (years)*				
	*Total*	*Boys*	*Girls*	*7th*	*8th*	*9th*	*10th*	*≤12*	*13*	*14*	*15*	*≥16*
Respondents	3485	1726	1737	1127	858	956	539	590	966	956	755	216
**Hookah use status**												
Ever smoked hookah	9.3 (7.6–11.4)	10.3 (8.2–12.7)	8.5 (6.4–11.2)	5.1 (3.7–7.1)	6.9 (4.4–10.6)	13.0 (10.1–16.5)***	14.2 (11.1–18.1)***	5.5 (3.6–8.4)	5.6 (3.6–8.6)	10.7 (8.6–13.2)***	11.7 (9.2–14.9)***	18.2 (13.9–23.5)***
Currently smoking hookah	0.9 (0.5–1.6)	0.8 (0.4–1.3)	1.0 (0.5–2.0)	0.4 (0.2–1.0)	0.6 (0.4–0.9)	1.2 (0.5–2.9)*	1.8 (0.9–3.5)*	0.4 (0.1–1.5)	0.2 (0.1–1.1)	1.3 (0.7–2.3)*	1.4 (0.7–2.7)*	2.0 (0.7–5.7)
Wanted to quit smoking hookah	1.5 (1.0–2.3)	1.8 (1.1–2.9)	1.2 (0.7–2.1)	0.4 (0.2–1.1)	0.9 (0.5–1.8)	2.1 (1.3–3.2)	3.1 (1.5–6.3)	1.3 (0.5–3.0)	0.3 (0.1–1.1)	1.4 (0.8–2.4)	2.7 (1.4–5.1)	2.9 (1.0–7.5)
**Perceptions of harm**												
Think that hookah is definitely not harmful	5.2 (4.2–6.3)	6.9 (5.5–8.7)	3.3 (2.6–4.4)***	4.1 (2.8–5.8)	3.7 (2.4–5.8)	7.0 (5.2–9.5)**	6.5 (4.0–10.3)*	4.1 (2.7–6.2)	3.8 (2.4–5.9)	5.3 (4.0–7.0)	6.9 (4.8–9.8)*	6.9 (3.6–12.7)*
Think that hookah is probably not harmful	7.5 (6.4–8.4)	8.4 (7.0–10.0)	6.7 (5.2–8.7)	5.4 (4.0–7.1)	9.7 (7.5–12.4)***	8.8 (6.8–11.3)**	5.7 (4.0–8.0)	4.7 (3.1–7.0)	8.6 (7.0–10.5)**	8.6 (6.3–11.5)**	6.8 (5.1–9.0)	7.7 (5.3–10.9)
Think that hookah is possibly harmful	28.8 (26.9–30.7)	25.8 (23.7–28.1)	31.9 (28.9–35.0)***	25.0 (22.8–27.4)	32.0 (29.0–35.1)***	28.5 (25.1–32.2)	29.9 (24.2–36.4)*	24.2 (20.2–28.7)	29.7 (26.1–33.4)*	29.6 (26.4–33.0)*	30.5 (26.9–34.3)**	25.8 (18.9–34.2)
Think that hookah is definitely harmful	58.5 (56.0–61.0)	58.9 (56.1–61.6)	58.0 (54.1–61.9)	65.6 (62.6–68.4)	54.6 (51.4–57.8)***	55.7 (51.6–59.7)***	57.9 (51.8–63.7)**	67.0 (62.1–71.5)	57.9 (54.4–61.3)***	56.6 (52.9–60.2)***	55.8 (51.8–59.8)***	59.7 (50.2–68.5)

Data are either given as count (n) or % (95% CI).

* p<0.05,

** p<0.01,

*** p<0.001.

Similar changes were noted in the group of daily hookah smokers. There are more hookah smokers in the 9th and 10th grades than in 7th grade (1.8% and 1.2% vs 0.4%; p<0.05), while current hookah smoking was higher among students aged 14 and 15 years compared to those aged 12 years (1.3% and 1.4 vs 0.4%; p<0.05).

There are gender differences in adolescents’ opinions about the dangers of hookah smoking. With boys more likely than girls to report that hookah smoking is definitely not harmful (6.9% vs 3.3%; p<0.001), while one in three girls versus one in four boys considers hookah to be probably harmful (31.9% vs 25.8%; p<0.001).

Doubt about the dangers of hookah smoking decreases with age. The percentage of students who consider hookah smoking as ‘definitely not harmful’ increased with age (7.0% in 9th grade and 6.5% in 10th grade, compared to 4.1% in 7th grade, with p<0.01 and p<0.05, respectively). The percentage of students who considered hookah smoking ‘definitely harmful’ is higher among 7th grade students (65.6%) compared to the higher grades (54.6% in 8th grade, 55.7% in 9th grade and 57.9% in 10th grade, with p<0.001, p<0.001 and p<0.05, respectively).

## DISCUSSION

This national survey provides the first data of the prevalence of hookah smoking among children and adolescents in Belarus and allows us to conclude that the growing popularity of waterpipe tobacco smoking dictates the need for timely recommendations on waterpipe smoking policy. The present study showed that boys more than girls were ever hookah smokers; however, the gender difference was not significant. There are gender differences in adolescents’ opinions about the dangers of hookah smoking.

There is a common misconception among young people about the relative safety of hookah smoking – the belief that because tobacco smoke travels through water, it makes hookah smoking less harmful than smoking cigarettes. This in turn contributes to its growing popularity and acceptability^6^. The belief about the dangers of hookah smoking is shared by only half of the students surveyed (58.5%), without any special differences in terms of gender.

GYTS results allow us to compare the prevalence of hookah smoking between Belarusian adolescents and their equals in age in other countries. In many countries, more young people use other tobacco products than smoke cigarettes. Waterpipe tobacco is highly addictive, and its use in the European Region is increasing rapidly, especially among young people and women^7^.

The European Region has some of the highest rates in the world, with overall rates of use of tobacco products other than cigarettes (including waterpipe tobacco) of 14% among boys aged 13–15 years (compared to 7% cigarette use) and 9% among girls aged 13–15 years (compared to 2% cigarette use). These data are similar to the Belarussian data we obtained from the GYTS analysis. The data obtained, showed that we are in the middle of the scale. Underlying this increase is the misperception that tobacco products such as waterpipe tobacco are less harmful to health than smoking cigarettes. Waterpipe tobacco is not a safe alternative to cigarettes.

## CONCLUSIONS

During adolescence, it is valuable and relevant to raise awareness about the dangers of tobacco and hookah smoking via the inclusion of health promotion aspects in educational programs to raise awareness of children and adolescents about the dangers of tobacco products.

Summing up, the data we obtained dictate to put in place a broad range of strategies needed to reverse the accelerating trend of hookah use in Belarus.

## Data Availability

The data supporting this research are available from the authors on reasonable request.
